# Perieccrine and pericapillary calcification in calciphylaxis

**DOI:** 10.12861/jrip.2015.03

**Published:** 2015-03-01

**Authors:** Christina Dookhan, Luis M Ortega, Ali Nayer, Jeong Hee Cho-Vega

**Affiliations:** ^1^Department of Internal Medicine, University of Miami Miller School of Medicine, Miami, FL, USA; ^2^Division of Nephrology and Hypertension, Allegheny General Hospital, Temple University School of Medicine, Pittsburgh, PA, USA; ^3^Division of Nephrology and Hypertension, University of Miami Miller School of Medicine, Miami, FL, USA; ^4^Department of Pathology, Dermatopathology Division, University of Miami Miller School of Medicine, Miami, FL, USA

**Keywords:** Calciphylaxis, Perieccrine calcification, Pericapillary calcification

***Implication for health policy/practice/research/medical education*:**

Calciphylaxis, also known as calcific uremic arteriolopathy, is characterized by ischemic tissue necrosis secondary to an obliterative vasculopathy. On histological examination, small and medium-sized arteries demonstrate medial calcification, intimal hyperplasia and thrombosis. However, vascular pathology may not be evident when small skin biopsies are examined. Subtle stippled perieccrine calcification revealed by calcium staining (von Kossa) is highly specific for calciphylaxis and can aid in the histological diagnosis of calciphylaxis when vascular calcification is not evident on small skin biopsies.

A 44-year-old Hispanic man presented with painful feet and penis. The past medical history was notable for end-stage renal disease, type 2 diabetes mellitus, hypertension, and peripheral vascular disease. The patient was on peritoneal dialysis. Medications included insulin, nifedipine, losartan, hydralazine, aspirin, gabapentin and calcium acetate. Physical examination revealed dusky red-purple discoloration of the left forefoot with a small ulcer on the great toe as well as dry gangrene of the right third and fourth toes and penis (Figure 1A-D). Laboratory tests demonstrated leukocytosis, anemia, hypoalbuminemia, vitamin D deficiency and secondary hyperparathyroidism (calcium 7.8 mg/dl, phosphate 10.7 mg/dl, parathyroid hormone 226 pg/ml). Cryoglobulins, lupus anticoagulant, and antibodies against nuclear antigens, myeloperoxidase, proteinase 3, hepatitis B and C viruses were not detected. Plain radiographs revealed diffuse vascular calcification of the feet. Computerized tomographic angiography demonstrated severe diffuse arterial calcification of the lower extremities. Echocardiography demonstrated preserved ejection fraction and no atrial septal defect. Skin biopsies showed epidermal and subcutaneous fat necrosis. No vascular calcification was noted in skin biopsies stained with hematoxylin and eosin (Figure 1E). However, calcium staining (Von Kossa) revealed subtle and stippled pericapillary and perieccrine calcification (Figure 1F,G). Skin biopsy from the penis showed microvascular thrombosis and subtle pericapillary calcification. Hospital course was notable for worsening ischemia of the left foot requiring a below-knee amputation. In the amputated leg, several medium-sized blood vessels in the subcutis showed intimal hyperplasia and medial calcification (Figure 1H).

**Figure 1:**
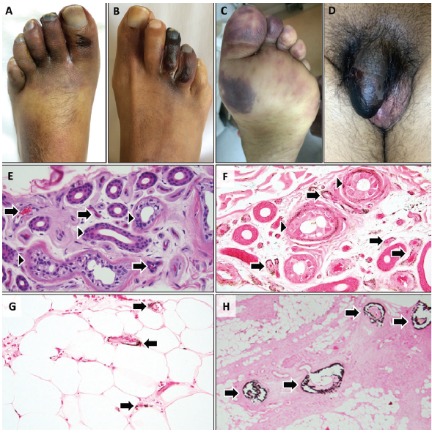
Perieccrine and pericapillary calcification in calciphylaxis. Severe ischemia of the feet manifested as dusky red-purple discoloration of the left forefoot as well as dry gangrene of the right third and fourth toes (A-C). Gangrene of the penis is shown (D). Skin biopsy stained with hematoxylin and eosin revealing eccrine glands (arrowheads) and capillaries (arrows) with no evidence of calcium deposition (E). Skin biopsy stained for calcium (Von Kossa) showing subtle stippled perieccrine (arrowheads) and pericapillary (arrows) calcification (F,G). Tissue section from the amputated leg stained for calcium (Von Kossa) showing medical calcification of several small arteries in the subcutis (arrows) (H).

Calciphylaxis, also known as calcific uremic arteriolopathy, is characterized by ischemic tissue necrosis accompanied by medial calcification, intimal hyperplasia and thrombosis of small and medium-sized arteries (1,2). The histological diagnosis of calciphylaxis can be challenging. Vascular medial calcification may not be evident in skin biopsies stained with hematoxylin and eosin. Perieccrine calcification revealed by calcium staining is highly specific for calciphylaxis (3). Pericapillary and perieccrine calcification revealed by Von Kossa stain can aid in the diagnosis of calciphylaxis when frank vascular medial calcification is not evident on routinely processed skin biopsies. In addition, the extent of calcification on skin biopsies may not correlate with the severity of calciphylaxis.

## Authors’ contributions

All authors wrote the manuscript equally.

## Ethical considerations

Ethical issues (including plagiarism, misconduct, data fabrication, falsification, double publication or submission, redundancy) have been completely observed by the authors.

## Conflict of interests

The authors declared no competing interests.

## Funding/Support

None.
